# Green synthesis of gold nanoparticles in Gum Arabic using pulsed laser ablation for CT imaging

**DOI:** 10.1038/s41598-022-14339-y

**Published:** 2022-06-22

**Authors:** Elham Mzwd, Naser M. Ahmed, Nursakinah Suradi, Saleh K. Alsaee, Abeer S. Altowyan, Munirah A. Almessiere, Ahmad Fairuz Omar

**Affiliations:** 1 School of Physics, Universiti Sains Malaysia (USM), 11800 Penang, Pulau Penang Malaysia; 2 Department of Physics, College of Science, Princess Nourah bint Abdulrahman University, P.O. Box 84428, Riyadh, 11671 Saudi Arabia; 3 Department of Physics, College of Science, Imam Abdulrahman Bin Faisal University, P.O BOX 1982, Dammam, 31441 Saudi Arabia; 4 Department of Biophysics, Institute for Research & Medical Consultations (IRMC), Imam Abdulrahman Bin Faisal University, P.O. Box 1982, Dammam, 31441 Saudi Arabia; 5 Department of medical instrumentation engineering, Dijlah university college, Baghdad, Iraq; 6 The University of Mashreq, Research Center, Baghdad, Iraq

**Keywords:** Optical physics, Quantum optics, Nanoscale materials, Computed tomography

## Abstract

Laser ablation synthesis in liquid solution (PLAL) is a green technique that allows for the physical formation of nanomaterials. This study indicates the preparation of stable gold nanoparticles (AuNPs) in Gum Arabic (GA) solution via laser ablation as a CT contrast agent. The optical properties were achieved using the absorption spectroscopic technique whereas the morphology and size distribution were investigated by TEM and ImageJ software. TEM image shows greater stability and spherical shape of GA-AuNPs with smaller size at 1.85 ± 0.99 nm compared to AuNPs without GA. The absorption spectrum of pure AuNPs has a lower absorption peak height in the visible range at λ = 521 nm, while the spectrum of GA-AuNPs has a higher plasmon peak height at λ = 514 nm with a blue shift towards lower wavelengths. The concentration of GA that dissolved in 10 mL of DI water via laser ablation is set at 20 mg. Increasing the number of pulses has only a minor effect on particle size distribution, which remains tiny in the nanometer range (less than 3 nm). For energies greater than 200 mJ, there is a blue shift toward shorter wavelengths. As the concentration of GA-AuNPs increases, the CT number is also increased indicating good image contrast. It can be concluded that there is a positive and significant influence of GA as a reducing agent for AuNPs, and a contrast agent for CT imaging which highlights its superiority in future medical applications.

## Introduction

Nanomaterials are currently present as a powerful tool and active area in research studies^1,2^ because of their size-specific^3^ and unique properties^4^. “Nano” is a prefix denoting for ten power to minus ninth which is a nanometer-scale^5^. Nanoparticles are particles with a diameter of fewer than 100 nm^2,6^. Metal nanoparticles (NPs) are utilized in many applications like medicine^4,7^, biosensing^8,9^, biomedical sciences, cosmetics, food, and electronics, creating impressive gains in each field^4^.

Gold nanoparticles (AuNPs) are extensively studied in several applications from material to medical science^10–13^ due to some unique qualities^14,15^; such as nontoxic, easy availability with controlled size and shape^6^, higher particle reactivity, surface modification capability, as well as high optical qualities^16^. As a result of the localized surface plasmon resonance (LSPR), gold nanoparticles demonstrate a red or purple colour nearly around a wavelength of 530 nm^11,12^. The properties of AuNPs can be modified by controlling their size, shape, and surface modification via synthetic processes.

Surface functionalization plays an essential role in the stability and hydrophilicity of the synthesized nanoparticles. The functionalization is the introduction of the ligands to modify the surface of NPs and prevent their colliding to use them in suitable applications^17^. The hydrophilic molecule is the compound which tends to attract the water^18^. Functionalization can be formed by adding an agent to the interactions during synthesizing the NPs^19^. One essential aspect that should take into consideration during producing the NPs is the interaction between the functionalization and the surface of the media where the NPs are synthesized. The polarization and ionic interaction between the functionalization and media could cause the instability of the NPs by creating aggregation of the particles^20^. Moreover; functionalization determines and controls the hydrophilicity of the NPs^21^.

Pulsed laser ablation in liquid media (PLAL) is currently recognized as a “green” physical alternative to conventional chemical methods^22,23^. The method includes the ablation of a solid target with strong laser rays, which results in the ejection of the target's constituents and the production of nanomaterials^24^. Advantages of this method are inexpensive^13,25^, the high purity of the nanomaterial^23,26^, nontoxic^4^, the material variety, and permitting the colloids to be handled safely and stably^26^. By this technique, AuNPs can be produced even in the absence of reducing agents. However, the size distributions of the AuNPs tend to be broadened due to the agglomeration and ejection of large fragments during laser ablation making them unstable^24^. To eliminate particle aggregation, a suitable stabilizer should be added^27^, to prevent particles from getting closer to each other^14^.

Researchers have used many reducing agents such as sodium citrate, gallic acid, hydrogen peroxide etc.^28^. However, the usage of citric acid, sodium borohydride (NaBH4), polyethene glycol (PEG), hexadecyltrimethylammonium bromide (CTAB), showed to be toxic^6^, harmful, irritating^29^, flammable, and hazardous to the environment^30,31^. Therefore, green synthesis methods were introduced recently, where chemical reducing agents are being replaced by plant extracts, bacteria, yeasts, fungi, and enzymes^29^.

One commonly used as a green stabilizer, and a reducing agent is Gum Arabic (GA) which is produced by Acacia Senegal trees^27,32^. GA is a polymeric material that is mostly composed of a long chain of glycoprotein, and polysaccharides, as well as greater amounts of magnesium, potassium, and calcium salts with a molecular formula of [C_15_H_20_NNaO_4_]^33^. GA atoms have carboxyl and amine groups that effectively bind to a nanoparticle's surface which enhances colloidal stability by creating steric repulsion among particles due to its great polysaccharides. The presence of a carboxyl functional group leads to an increase in the chemical reactivity and connects the compound to adjacent molecules^15^. GA coating can provide high stability of AuNPs with smaller size, excellent properties and no significant adverse or toxic actions^34^. These properties make GA-AuNPs suitable for biomedical applications such as imaging contrast agents, targeting, drug and gene delivery^15,35–37^.

Recently, computed tomography (CT) is the most widely diagnostic approach that used X-ray in 3D anatomic images with high-resolution^38,39^. However, the ability to distinguish between surrounding tissues is a serious problem due to the tiny differences in the X-ray attenuation for the soft tissues^38,40^. This limitation is overcome by using iodine contrast, but it has harmful influences on the human body. Therefore, nanomaterials are considered as alternative contrasts. One of the most nanomaterials used as contrast is the AuNPs because of their unique properties. The AuNPs have to be coated with a stabilizer to prevent aggregation and improve their stability. In this study, the Gum Arabic will be used as a coating for AuNPs. Gum Arabic coated gold nanoparticles are promising as a new contrast agent for CT imaging^41^. Katti’s group created AuNPs coated with GA as good contrast agents for computed tomography (CT)^42^. Chen et al. also synthesized GA- Au-NPs through heating in the absence of reducing agents and thus resulting in stable and high ionic strength solutions^35^.

To the best of our knowledge, there is a lack of studies that investigate the ability of AuNPs coated by GA, which is synthesized by pulsed laser ablation, as CT contrast agents. The main aim of this study is to use GA-AuNPs as a contrast agent in CT imaging and investigate their properties. The GA-AuNPs are synthesized by PLAL with different laser energies, GA concentrations and number of laser pulses and their effects on particle size and production are investigated. The morphological and optical properties of the studied nanoparticles are investigated using TEM, and UV Vis. The effects of the GA-AuNPs concentrations on CT numbers were investigated by the CT scanner.

## Methodology

### Materials

A rounded gold plate (99% purity, Z = 79) with 0.5 mm thickness, a diameter of 5 mm, and 0.5 g is used as a laser target. 20 mL acetone (≥ 99.5%) from Sigma Aldrich is used to clean the gold target. DI water (Arium® pro-Ultrapure Water System with a conductivity of 00.055 μS/cm compensated to 25 °C-school of physics) and Al-Noor Gum Arabic (100% pure) from Acacia Senegal are utilized to prepare GA solutions as a medium. High-quality DapurDesa agar–agar strips 25 mg are used to reconstruct the CT phantoms.

### Apparatus and instruments

Branson with an ultrasonic bath (professional ultrasonic cleaner) was used to purify the gold target from the contamination at the solid-state lab-school of physics, USM. Pulsed laser ablation in liquid technique (PLAL): Q-switched Nd: YAG (Neodymium—Doped Yttrium Aluminum Grant; Nd: Y3A15012) with wavelength at 1064 nm was utilized to synthesise the nanoparticles in GA solutions. Atomic Absorption Spectrometer (AAS-Perkin Elmer, Analyst 400, 2014) was hired to acquire the concentration (M) of GA-AuNPs which measures the number of atoms in a sample in the parts-per-billion range (ppm) at Makmal Unit Perkhidmatan Analysis-School of Chemical Science, USM. AAS measurements were conducted two times for each sample and the average value was taken. The absorption spectrum was conducted using a UV–Vis Jaz spectrometer with 360 to 2000 nm of the tungsten-halogen emitter to explore the optical properties at the Engineering Laboratory, School of Physics, USM. ZEISS LIBRA®120 high-resolution transmission electron microscope (point-to-point resolution 0.34 nm) at MICROSCOPY UNIT—School of biology, USM was employed to investigate the structure and morphology of nanoparticles. Toshiba Aquilion with 64-slice CT scanner with X-ray voltage at 80 kVp and the anode current of 50 mA via head window (L: 40HU, W:120 HU) was used to evaluate the GA-AuNPs phantoms at Gleneagles Hospital Penang.

### Preparation of Gum Arabic solutions

Various Gum Arabic (GA) quantities are weighted at (15, 20, 30, and 40 mg) as in supplementary file Fig. S1a. These GA volumes have been dissolved in 10 mL DI water under a magnetic stirrer with the lowest rate of around 200 rpm for 30 min at a temperature of 55 °C.

### Synthesis of gold nanoparticles by laser ablation

Firstly, the gold target was cleaned by Branson with an ultrasonic bath (professional ultrasonic cleaner) and washed with 20 mL acetone to get rid of organic contamination for 15 min at 40 °C temperatures. Then, it was rinsed with deionized water. After that, AuNPs were produced by laser ablation of a gold plate in DI water in the absence and presence of GA. The gold plate was positioned at the bottom of a glass vase containing 10 mL of GA aqueous solution, and was exposed to the output of the fundamental (1064 nm) wavelength of Q-switched Nd: YAG laser performing at 10 Hz, 1000 mJ, and 1000 pulses as seen in Supplementary file Fig. S1b. The formation of gold nanoparticles was performed to find out the factors that influence the size, and production of AuNPs through main three directions^43^ which are; concentration of Gum Arabic at 15, 20, 30, 40 mg, number of laser pulses at 200, 500, 1000, 2000 pulses, and laser power at 50, 100, 200, 500, 1000, 2000 mJ/pulse.

### Preparing samples for characterization

After the production of NPs, the samples are taken for characterization. The spectroscopic measurements were carried out by positioning NPs samples separately on the cuvette holder in which DI water was used as a reference. The origin pro8 program was employed to draw the absorption spectra. Through HR-TEM, a small drop of the sample was put on a copper grid with a carbon coating and let dry for 20 min at room temperature. After that, the grid was taken to the HR-TEM device and the images were acquired. Image J software was used to analyze the HR-TEM images to measure the size of the nanoparticles. The distribution of the GA-AuNPs size was taken by observing 20 to 70 particles. Later, Origin Pro 8 software was performed to get the Gaussian histogram for size distribution.

### In vitro CT imaging

To test GA-AuNPs /AuNPs for CT imaging, agarose gels were used to fabricate CT phantoms. Agar strips weighing 1 g were dissolved in 50 mL DI water in a glass beaker. The solution was heated with a magnetic stirrer until it was boiling, then cooled to 100 °C for 20 min. 20% of the agar solution was combined with 1 mL of GA-AuNPs at concentrations of 55, 28, 14, and 7 ppm, and poured into 2 mL vials. The samples then were allowed to dry for two hours before being placed in the refrigerator for 24 h to harden. The same procedures are repeated for AuNPs phantoms at concentrations of (0, 6, 11, 21, 42) ppm.

A Toshiba Aquilion 64 slice CT scanner was used for the imaging in which the brightness of the photos indicates signal strength. The CT imaging parameters include the X-ray voltage of 80 kV and an anode current of 50 mA via the head window (L: 40 HU, W: 120 HU) where the scan is taken two times for each sample and the average was taken. CT images were characterized by the DICOM software that depends on the signal intensity to calculate HU as in [Eq. (1)]. FOV and slice thickness were set at 320 mm, and 3 mm, respectively.

1 $$HU=\frac{\mu -{\mu }_{water}}{{\mu }_{water}} \times 100$$

where HU = Hounsfield Unit, μ is the linear X-ray attenuation coefficient of the substance and μ_water_ is the linear attenuation coefficient of water.

## Results and discussion

### Synthesis of AuNPs and GA-AuNPs

**Figure 1:**
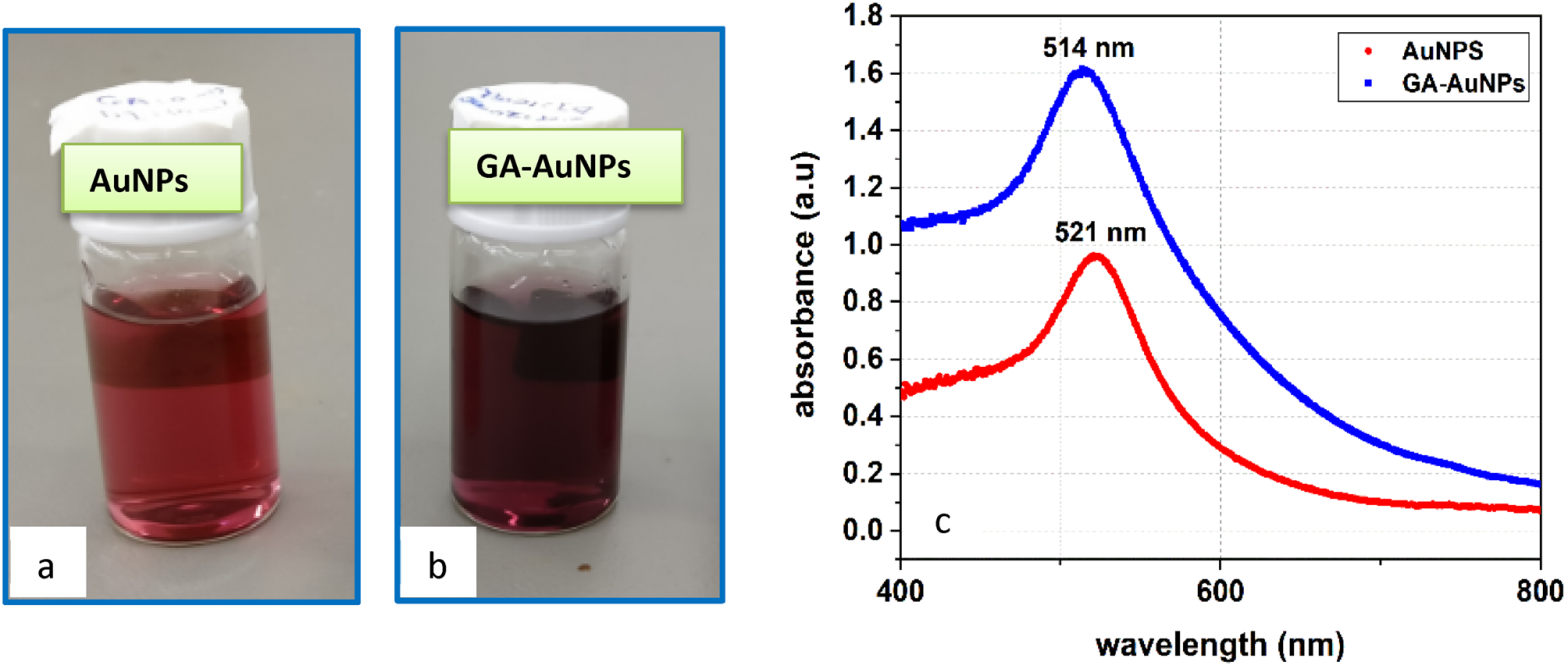
Optical images of (**a**) AuNPs at GA = 0, (**b**) GA-AuNPs at GA = 20 mg, and (**c**) Absorption specrtum for both of them.

Gold nanoparticles are synthesized via laser ablation in DI water in the absence, and presence of GA under the same parameters separately. After seconds of ablation, the colourless solution gradually changes to light red for the sample AuNP, and dark purple for GA-AuNPs as in Fig. 1a and b. This change in colour due to the oscillation of the conduction band electrons in the nanoparticles^44^ is known as localized surface plasmon resonance (LSPR) which occurs when the frequency of the light photons matches the inherent frequency of the nanoparticles’ surface electrons^45^ creating the reddish tones^46^. The colour intensity of the solution is relevant to the rise of the nanoparticles concentrations in the colloid^45^. From the AAS measurements, it is observed that the GA-AuNPs have higher concentrations (M = 54.57 ppm) compared to AuNPs concentration (M = 41.42 ppm). The high concentration of the functionalized particles (GA-AuNPs) is due to the coating by Gum Arabic which makes each particle apart from the others, so the number of individual particles will be higher. However, for the naked particles (AuNPs), some particles aggregate to form a particle with higher size, thus the number of particles will be reduced.

In Fig. 1c, the absorption spectrum of GA-AuNPs has a maximum height of plasmon peak in the visible range at λ = 514 nm which signifies the successful synthesis of AuNPs with high concentration. On the other hand, the spectrum of AuNPs has a lower height of absorption peak at λ = 521 nm and a little red shift towards higher wavelengths. When the diameter of nanoparticles is declined, the electrons escape from the surface of nanoparticles and conduct to the affinity energy level of the substrate, which means that the density of electrons decreases and the frequency of free-electron plasma (wp) decreases as well. Because the frequency of the localized surface plasmon resonance is proportional to (wp), so the absorption peak moves to the left of the original one^47^. According to Yokoyama (2018), the maximum light absorption wavelength for 15 nm particles is 525 nm, however, it is increased by roughly 50 nm for 45 nm particles^5^.

**Figure 2:**
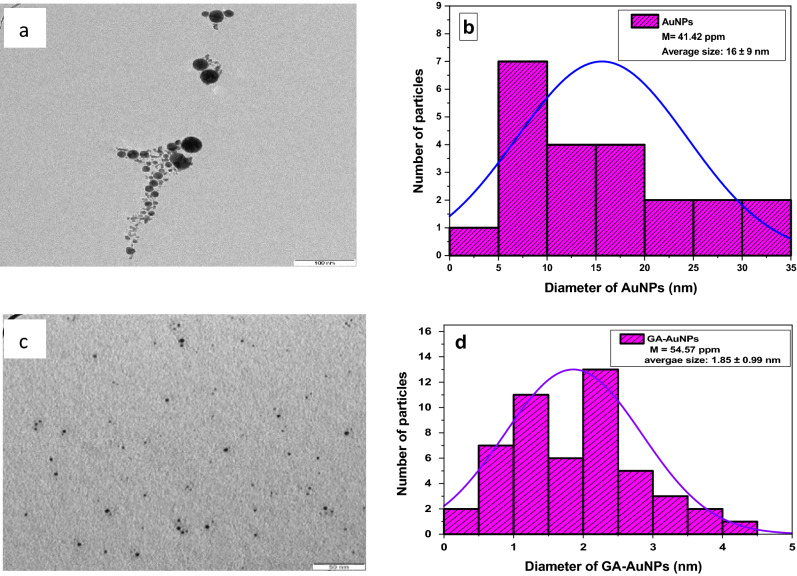
HR-TEM images of (**a**) AuNPs, and (**c**) GA-AuNPs; size particle distribution of (**b**) AuNPs, and (**d**) GA-AuNPs.

Gum Arabic is employed to wrap the gold nanoparticles and protect them from aggregation^48^. The gum acacia network's hydroxyl groups hold the nanoparticles together through hydrogen bonding, allowing them to remain apart and offering nanoparticle stability and smaller size^49^. Figure 2-a shows the morphology of the TEM image of AuNPs (M = 41.42 ppm) with spherical shapes and a lot of aggregations groups. It is observed that the size of the prepared nanoparticles is about (16 ± 9) nm as shown in the size distribution in Fig. 2b. Furthermore, Fig. 2c represents the TEM image of spherical particles GA-AuNPs (M = 54.57 ppm) in which there are no significant aggregations. Figure 2d indicates the size distribution of GA-AuNPs which is shifted to smaller values in particle size at 1.85 ± 0.99 nm^15^. Our results are in good agreement with Barros et.al., findings of spherical GA-AuNPs with average size (5.4 ± 2.1 nm) and aggregation groups of (16.2 ± 6.1) nm for the bare-AuNPs^15^. One crucial parameter for the nanoparticles is the stability of nanostructure, particularly in their morphology, size, distribution, and surface characteristics^50,51^. According to literature, the small size is an indication of particle stability^52^, as well as the clusters and aggregations, are signs of particle instability^53^. Thus results of this work provide more stability with a smaller size and no aggregation of GA-AuNPs compared to the study^15^ findings.

### Optimization synthesis parameters on size and production of GA-AuNPs

This research has been concerned with the study of the GA-AuNP production and size as a function of the various parameters of medium and laser paying attention to the following effects; effect of solvent concentration (Gum Arabic), the influence of laser pulses number per second, and laser energy (mJ) effects.

**Figure 3:**
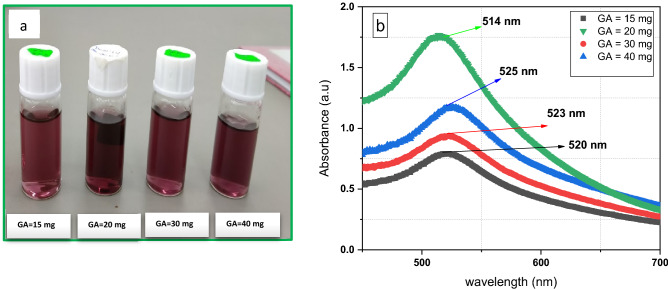
(**a**) Optical images, and (**b**) the absorption spectrum of GA-AuNPs at different concentration of GA (15, 20, 30, 40 mg).

GA-AuNPs were synthesized with different concentrations of GA at (15, 20, 30, 40 mg). After a while of ablation, the colourless solution is transformed into light and dark purple-red according to the amount of GA. The most darkly colour solution is at 20 mg of GA at a concentration of (M = 54.57 ppm) as in Fig. 3a. The concentrations of GA ranged from (15 to 40) mg, which can be divided into three regions depending on the similar behaviour on their absorption spectrum. The three regions are low concentrations (15) mg, middle region (20 mg), and high concentrations (30–40) mg. In Fig. 3b at GA = 15 mg, the height of the absorption peak is low at λ = 520 nm. Obviously, with increasing the amount of GA to 20 mg, there is a sudden increase in the peak height at λ = 514 nm; indicating the increase in the concentration of GA-AuNPs. However, there is a blue shift in plasmon peaks towards lower wavelengths. Further raise in GA concentrations to 30, 40 mg provide a slight increase in the plasmon peak height compared to 15 mg, but it is lower than 20 mg, which has the highest peak. These findings are consistent with those obtained from AAS measurements.

At lower GA concentration (15) mg, there is a red-shift extinction where the particle aggregation and size distribution of GA-AuNPs is larger due to the insufficient protection of GA. At the concentration of (20 mg), there is a sudden blue shift towards the lower wavelength that indicates the mean diameter is reduced due to the good protection of GA that stabilizes the particles^35^. For high GA concentrations (30–40) mg, there are redshifts again that increase slightly with increasing the concentration of GA due to the higher intermolecular force of GA on the target may inhibit AuNPs scattering^54^. Thus, insufficient or excess GA is not recommended for the stabilization of Au nanoparticles^35^. As a result, 20 mg of GA is chosen as an optimum amount for dissolving in 10 mL of DI water via laser ablation. Shahidi et al. synthesized GA-AuNPs by dissolving 15 mg of GA in 10 ml of DI water, but they gain a larger NPs size and their absorption peak has a redshift around 535 nm^54^. So, our method in coating AuNPs with Gum Arabic helps in size reduction of the gold particle by providing high environment stability compared to previous study^54^.

**Figure 4:**
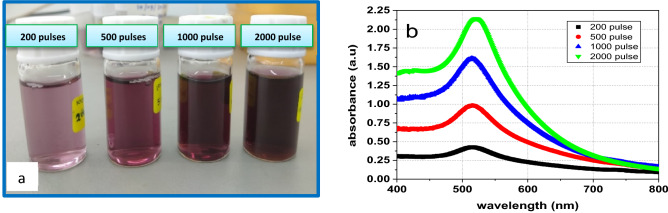
(**a**) Optical images, and (**b**) the absorption spectrum of GA-AuNPs at different number of laser pulses at (200, 500, 1000, 2000).

GA-AuNPs were synthesized with varying numbers of pulses at (200, 500, 1000, and 2000 pulses/s). In Fig. 4a; it is realized that at low pulses (200 pulses), the colour of GA-AuNPs is light red. However, when the pulses are increased to 500 pulses, the colour becomes lighter purplish-red, darker at 1000 pulses, and denser at 2000 pulses. The concentration for each solution is affirmed as in Table 1 in which the highest concentration is for 2000 pulses at 75.6 ppm.

**Table 1:** Concentrations of GA-AuNPs samples by AAS at (200, 500, 1000, 2000 pulses).

Number of pulses	Solution colour	Average concentration of GA-AuNPs (ppm)	Absorbance (a.u)	Absorption peak (nm)
200	Light pink	13.07 ± 0.0054	0.427	513.63
500	Light purplish-red	36.22 ± 0.0053	0.985	515.18
1000	Dark purplish-red	54.68 ± 0.0096	1.614	513.63
2000	Dense purplish-red	75.6 ± 0.0119	2.141	521.0

Figure 4b indicates that as the number of pulses increases, the plasmon peak height raised, indicating an increase in nanoparticle concentration, until the NPs reach their critical size, at which point the gold nanoparticles absorption coefficient is reduced and they can't absorb any more because they can't be fragmented again, resulting in a blue shift in the plasmon peak, indicating a decrease in the gold nanoparticles size^55^.

More nanoparticles are created as the number of laser pulses increases, and they cluster together near the laser focus. After a significant number of nanoparticles have been produced, a substantial number of them cover the target surface, resulting in a decrease in energy absorption and, as a result, a reduction in the ablation rate. Furthermore, predominantly produced nanoparticles absorb a considerable portion of the laser pulse energy, resulting in nanoparticle size reduction. The size reduction of NPs is caused by the absorption of heat, which raises the temperature of the particles, causing melting and cooling^56^. When the temperature of a gold particle reaches its boiling point, atoms and tiny particles are expelled by vaporization. As a result, particle sizes are reduced^57^.

**Figure 5:**
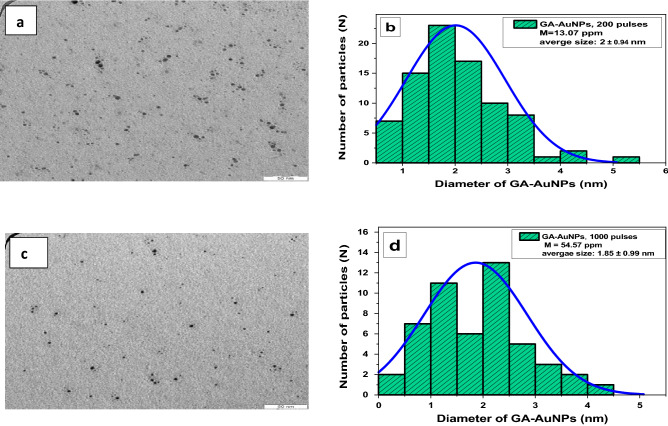
HR-TEM images of GA-AuNPs at (**a**) 200 pulses, and (**c**) 1000 pulses; size distribution histogram of GA-AuNPs at (**b**) 200 pulses, and (**d**) 1000 pulses.

TEM images are taken for 200 pulses (as low) and 1000 pulses (as high) to investigate the influence on the particle size and shape. Figure 5a and c indicate the spherical shape of nanoparticles and the average sizes are acquired via the best Gaussian fit of the size distribution curves^15^ which provides the average size of 2 nm at 200 pulses and 1.85 nm at 1000 pulses as seen in Fig. 5b and d respectively. The results reveal that increasing the number of pulses has only a minor effect on particle size distribution. Our results are in good agreement with^58^ which reported that by changing the laser pulses, the mean size of the particle is still in the nanometer region with less than 3 nm^58^. Therefore, changing the number of pulses influences only the concentrations of GA-AuNPs proportionally, however, there is no significant effect on nanoparticle size.

**Figure 6:**
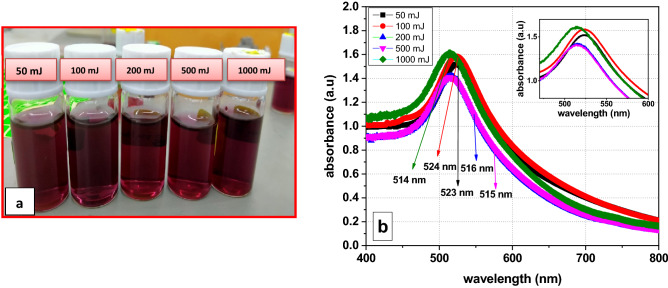
(**a**) Optical images, and (**b**) absorption spectrum of GA-AuNPs at different laser energy of (50, 100, 200, 500, 1000 mJ) under same other parameters.

By varying the laser energy at (50,100, 200, 500, 1000 mJ) as in Fig. 6a, it is noticed that the colour of all samples of GA-AuNPs is roughly the same dark purplish-red and no remarkable change in the colour or the concentrations of the particles by AAS measurements (M ~ 52–54 ppm). The energy of the laser ranged between (50–1000 mJ), and can be divided into two regions according to their similar properties on the absorption spectrum; low energies (50–100 mJ), and high energies (200–500–1000 mJ). At low energies (50, 100 mJ), Fig. 6b shows a slight increase in the height of absorption peak with raising the laser energy. Also, there is a little red shift towards higher wavelengths at (523 nm, and 524 nm) respectively. At these low energies, the laser power is absorbed in the solution resulting in material removal by reactive sputtering more than the direct laser ablation. So only a few amounts of the light reaching to the target. The plasma formation in the solution creates a cavitation bubble that expands and then collapses, driving highly energetic species into the target. In other words, the nanoparticles in the solution grow by attracting the small fragments. In this region, the average size of gold nanoparticles begins to increase ^55^.

**Figure 7:**
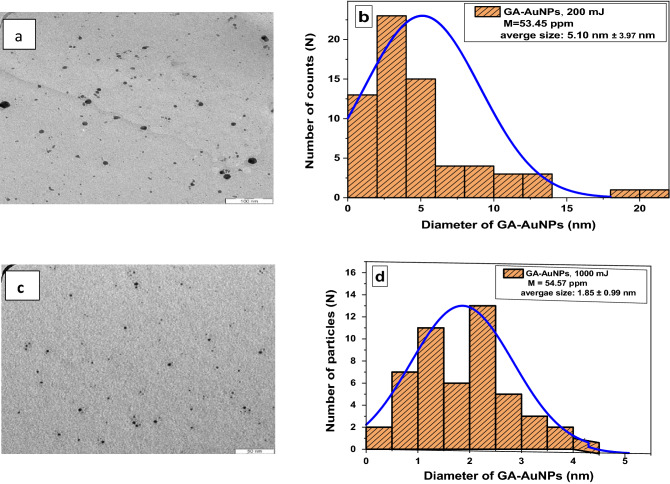
HR-TEM images of GA-AuNPs at (**a**) 200 mJ, and (**c**) 1000 mJ; size partictle distribution for GA-AuNPs at (**b**) 200 mJ, and (**d**) 1000 mJ.

At high laser energy (200, 500, 1000) mJ, as shown in Fig. 6b, the absorbance shows a little increase in the maximum height of the plasmon peak with increasing the laser energy, indicating no remarkable change in the concentration of gold nanoparticles. As the energy increase, the peak becomes narrower and there is a blue shift in the plasmon peak around ~ (1 nm). So, for high energies, as the energy increase, the size of the gold nanoparticles decreases only slightly. These results are matching as obtained from ASS and TEM images^55^. At these high energies, when the energy is raised gradually, the size distributions and average NP size are initially greater, but continuous laser irradiation of the solution may cause fragmentation of the Au-NPs in the solution, resulting in size reduction. Also, other factors such as fragmentation, boiling, and vaporization help to separate particles from the solid target^13,25^. Due to the quantum confinement influence, shifting to shorter wavelengths indicates the production of smaller nanoparticles^59^. Figure 7a and c represent the TEM images of the GA-AuNPs with an average particle size of 5.1 nm (Fig. 7b), and 1.85 nm (Fig. 7d) at 200 mJ and 1000 mJ; respectively.

**Figure 8:**
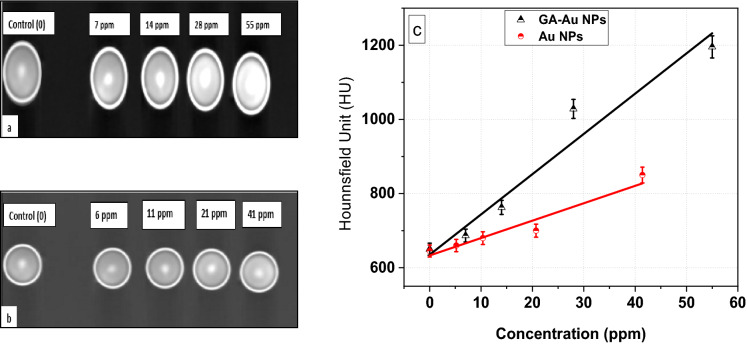
CT images for CT phantoms at different concentrations of (**a**) GA-AuNPs, and (**b**) AuNPs by DICOM software. (**c**) Linear dependence of HU on the GA-AuNPs and AuNPs concentrations.

The concentrations effects of GA-AuNPs and AuNPs on the CT number values were invistagted. At low concentration, the brightness is low, however, at high concentration, the brightness increases as shown in Fig. 8a and b for GA-AuNPs and AuNPs respectively. Compared to AuNPs, GA-AuNPs shows higher impacts on CT image causing the image to be more brightness due to the confinement of the GA-AuNPs. This effect proves that the X-ray attenuation is higher leading to better image resolution. Increasing the CT number values linearly as in Fig. 8c proves that AuNPs have been coated with GA and their stability increases which gives good contrast for CT imaging^38,54^. In CT images, the contrast is due to variation in the electron densities (attenuation coefficients) of various tissues. Gold has a high electron density, consequently, GA-AuNPs can be utilized as effective contrast agents in CT imaging^60^. Comparing to previous literature^61^, this study for synthesizing GA-AuNPs by PLAL as a contrast agent provides more brightness with a higher CT number as well as the synthesized method is an easier and green technique.

This study added new knowledge about the suitable concentration of GA which is (20 mg in 10 ml of DI) that can provide high NPs absorbance and blue shift of the absorption peak at λ = 514 nm. Also, we achieve a small size with no aggregation by providing high environment stability compared to some studies as in^54^. Furthermore, we highlight the effects in detail of laser parameters on GA-AuNPs concentration and size as well as the impact of the coating process around the prepared nanoparticles. These properties of GA-AuNPs inspire us to use them in CT imaging which achieved high CT numbers and great image quality.

## Conclusion

Synthesis of gold nanoparticles by the pulsed laser ablation technique in liquid was investigated. Based on our findings, the presence of Gum Arabic plays a significant influence on the size and production of the prepared nanoparticles. Compared to AuNPs, GA-AuNPs award smaller size and no aggregation because GA acts as an effective stabilizer that controls the production of the AuNPs and coating them. The concentration of GA impresses AuNPs features deeply. 20 mg of GA achieves a blue shift of the absorption peak at λ = 514 nm providing high stability and size reduction. Laser ablation parameters have a huge impact on GA-AuNPs’ properties. The number of laser pulses influences the nanoparticle size slightly and remains less than 3 nm. The laser energy also influences GA-AuNPs' qualities. Energies above 200 mJ provide great stability with small NPs sizes. GA-AuNPs concentrations affect the CT image quality. Increasing the concentration of GA-AuNPs to 54 ppm, enhanced the CT number and thus improved image contrast. According to our results, GA can be a superior tool for researchers to investigate its benefits in biomedical applications. We recommended the use of GA as a good contrast agent for medical imaging in the US, MRI as well as a good targeting agent for cancer therapy.

## Supplementary Information

Supplementary Information.

## Data Availability

The data that supports the findings of this study are available within the article.
