# Supplementary material for: Green synthesis of gold nanoparticles in Gum Arabic using pulsed laser ablation for CT imaging
Source: Sci Rep. 2022 Jun 22;12:10549. doi: 10.1038/s41598-022-14339-y (PMC9218112; doi:10.1038/s41598-022-14339-y)
Supplement: Supplementary file 1 — Supplementary Information. [file 41598_2022_14339_MOESM1_ESM.docx]

**Green Synthesis of Gold Nanoparticles in Gum Arabic Using Pulsed Laser Ablation for CT Imaging**

Elham Mzwd^1,2^, Naser M. Ahmed^1^, Nursakinah Suradi^1^, Saleh K. Alsaee^1,3^, Abeer S. Altowyan^4*^, Munirah A. Almessiere^5,6^, Ahmad Fairuz Omar^1^

^1^School of Physics, Universiti Sains Malaysia (USM), 11800 Penang, Pulau Penang, Malaysia

^2^Physics Department, Faculty of Education, Seiyun University, Hadhramout, Yemen

^3^Physics Department, Faculty of Science, Hadhramout University, Al-Mukalla 50512, Hadhramout, Yemen

^4^Department of Physics, College of Science, Princess Nourah bint Abdulrahman University, P.O. Box 84428, Riyadh 11671, Saudi Arabia.

^5^Department of Physics, College of science, imam abdulrahman bin faisal university, P.O BOX 1982, Dammam 31441, Saudi Arabia

^6^Department of Biophysics, Institute for Research & Medical Consultations (IRMC), Imam Abdulrahman Bin Faisal University, P.O. Box 1982, 31441, Dammam, Saudi Arabia

***Corresponding author:** [asaltowyan@pnu.edu.sa](mailto:asaltowyan@pnu.edu.sa)

1. **Experimental part**
2. **Synthesis of gold nanoparticles by laser ablation**

Fabrication of AuNPs and GA-AuNPs were carried out using pulsed laser ablation in liquid technique (PLAL): Q-switched Nd: YAG (Neodymium – Doped Yttrium Aluminum Grant; Nd: Y3A15012) with wavelength at 1064 nm as in Figure 1S below:

Gum Arabic


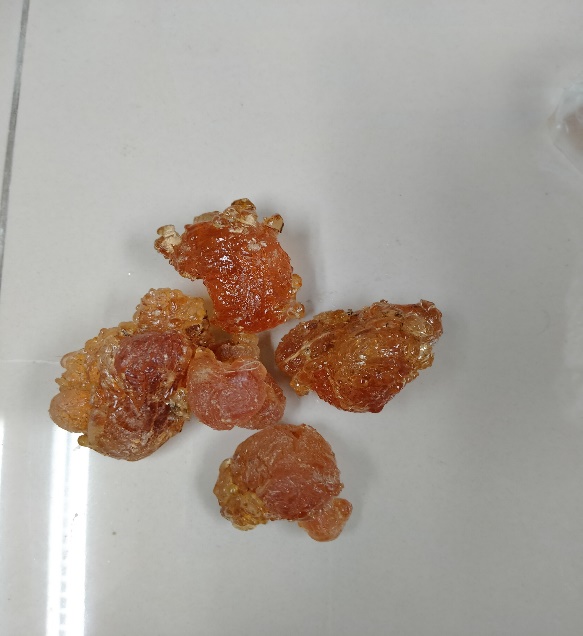


a


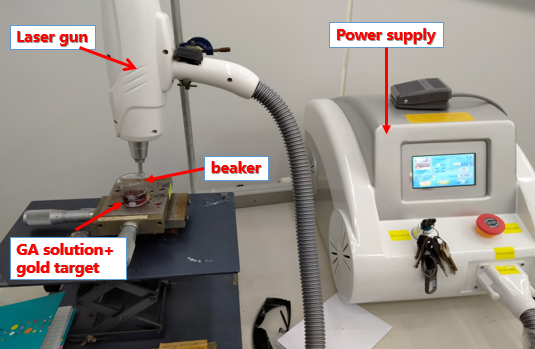


b

**Figure S1:** a) Gum Arabic, and b) The arrangment of pulsed laser ablation in liquid (PLAL).

1. **Atomic Absorption Sectrometer (AAS) measurements**

**2.1: Synthesis of AuNPs and GA-AuNPs:**

**Table S1:** Concentrations of AuNPs and GA-AuNPs by AAS measurements.

| **Sample** | **Solvent type** | **Concentrations of NPs in (ppm)** | | **Average Concentration (ppm)** |
| --- | --- | --- | --- | --- |
| AuNPs | DI water only | 41.30 | 41.55 | 41.42 ± 0.0177 |
| GA-AuNPs | DI+ GA:20 mg | 54.68 | 54.28 | 54.57±0.0247 |

**2.2: Different concentration of GA:**

**Table S2:** Concentrations of GA-AuNPs by AAS at different of GA (15, 20, 30, 40 mg).

| **Sample No.** | **Amount of GA**  **(mg)** | **Concentrations of GA-AuNPs in (ppm)** | | **Average concentration in (ppm)** |
| --- | --- | --- | --- | --- |
| 1 | 15 | 40.03 | 39.60 | 39.81± 0.0306 |
| 2 | 20 | 54.40 | 54.75 | 54.57± 0.0247 |
| 3 | 30 | 48.03 | 48.34 | 48.19±0.0214 |
| 4 | 40 | 48.70 | 50.53 | 49.61± 0.1292 |

**2.3: Different number of pulses:**

**Table S3:** Concentrations of GA-AuNPs samples by AAS at different number of pulses (200, 500, 1000, 2000 pulses).

| **Sample No.** | **Number of pulses** | **Concentrations of GA-AuNPs in (ppm)** | | **Average Concentration in (ppm)** |
| --- | --- | --- | --- | --- |
| 1 | 200 | 13.11 | 13.03 | 13.07 ± 0.0054 |
| 2 | 500 | 36.18 | 36.26 | 36.22 ± 0.0053 |
| 3 | 1000 | 54.62 | 54.75 | 54.68± 0.0096 |
| 4 | 2000 | 75.4 | 75.825 | 75.6 ± 0.0119 |

**2.4: Different laser power:**

**Table S‎4:** Concentrations of GA-AuNPs samples by AAS at different laser power (50, 100,200, 500, 1000, mJ)

| **Sample No.** | **Laser power**  **(mJ)** | **Concentrations of GA-GNPs in (ppm)** | | **Average concentration in (ppm)** |
| --- | --- | --- | --- | --- |
| 1 | 50 | - | - | - |
| 2 | 100 | - | - | - |
| 3 | 200 | 53.27 | 53.63 | 53.45± 0.0155 |
| 4 | 500 | 52.8 | 52.3 | 52.55± 0.0138 |
| 5 | 1000 | 54.62 | 54.75 | 54.68± 0.0096 |
